# “I felt like a lone ranger”: experiences of Australian families living with *KIF1A*-Associated Neurological Disorder

**DOI:** 10.1007/s12687-026-00908-5

**Published:** 2026-06-08

**Authors:** Kara Miwa-Dale, Kimberley Norman, Belinda Dawson-McClaren, Jeanette Harris, Wendy A. Gold, Trang T. Do, Simranpreet Kaur

**Affiliations:** 1 Department of Paediatrics, The University of Melbourne, Melbourne, VIC Australia; 2 Brain and Mitochondrial Research Group, Murdoch Children’s Research Institute, Royal Children’s Hospital, Melbourne, VIC Australia; 3 School of Primary and Allied Health Care, Monash University, Melbourne, VIC Australia; 4 Genomics in Society, Murdoch Children’s Research Institute, Melbourne, VIC Australia; 5 KIF1A Australia Foundation, Yatala, QLD Australia; 6 School of Medical Sciences, Faculty of Medicine Health, The University of Sydney, Sydney, NSW Australia; 7 Kids Neuroscience Centre, Kids Research, Children’s Hospital at Westmead, Westmead, NSW Australia; 8 Molecular Neurobiology Research Laboratory, Kids Research, Children’s Hospital at Westmead and the Children’s Medical Research Institute, Westmead, NSW Australia

**Keywords:** *KIF1A*-Associated Neurological Disorder (KAND), Rare disease, Childhood dementia, Qualitative research, Caregiver experiences

## Abstract

*KIF1A-*Associated Neurological Disorder (KAND) is a heterogeneous group of ultra-rare neurodegenerative conditions. Severe forms of KAND, classified within the broader category of childhood dementia, lead to progressive loss of motor, communication and cognitive skills with markedly reduced life expectancy. Although the disorder is genetically and clinically defined, little is known about how families experience diagnosis and ongoing care. To address this gap, we conducted the first empirical investigation into the lived experiences of families living with KAND. Fifteen semi-structured interviews were conducted with 16 parents of individuals with KAND; one interview also included an individual living with KAND. Data were analysed using inductive content analysis. Families described healthcare journeys shaped by frustration and uncertainty, with early concerns frequently dismissed, diagnoses delayed, and access to clinicians with relevant expertise limited. These challenges were compounded by the financial, administrative, psychological and relational strain associated with the complex care needs of KAND. Families reported unmet support needs, including limited access to condition-specific information, difficulties navigating formal support systems, and uncertainty surrounding long-term care planning. In response to these gaps, parents emphasised the need for clearer diagnostic pathways, coordinated multidisciplinary care, and a centralised network of informed healthcare professionals. In the absence of formal support, families relied heavily on peer support networks for information and psychosocial connection. By identifying previously undocumented gaps in rare disease service delivery and support, these findings highlight opportunities to improve clinical genetics practice, strengthen support pathways, and guide future research aimed at enhancing quality of life for individuals with KAND and their families.

**Supplementary Information:**

The online version contains supplementary material available at 10.1007/s12687-026-00908-5.

## Introduction

*KIF1A*-Associated Neurological Disorder (KAND) comprises a group of ultra-rare neurodegenerative conditions caused by pathogenic variants in *KIF1A*, which encodes a neuron-specific motor protein essential for axonal transport (Boyle et al. 2021; Kaur et al. 2020). Disruption of KIF1A-mediated transport along microtubules impairs neural communication, resulting in widespread dysfunction of the central and peripheral nervous systems (Nemani et al. 2020).

KAND is characterised by a broad phenotypic spectrum, including severe intellectual disability, language impairment, hypotonia, spasticity, ataxia, drug-resistant epilepsy, cerebellar atrophy, cortical visual impairment, optic nerve atrophy, peripheral neuropathy, and neuropsychiatric features (Vecchia et al. 2022). Only around 600 cases have been reported worldwide (KIF1A.ORG 2025), although the prevalence is likely to be a significant underestimate due to limited availability of genetic testing, frequent misdiagnosis as cerebral palsy and under-recognition in clinical practice (Boyle et al. 2021). Disease onset, progression and prognosis vary substantially, reflecting the diversity of pathogenic *KIF1A* variants (Boyle et al. 2021), and range from severe infantile onset neurodegeneration with early mortality (Nair et al. 2023) to more mild impairment allowing survival into adulthood (Akkus et al. 2025).

There are currently no clinically established targeted therapies for KAND, with management focused on supportive and symptomatic care (Lin et al. 2025). Although antisense oligonucleotide therapy has emerged as a potential disease-modifying treatment (Ziegler et al. 2024), it remains experimental and is inaccessible to most families, with treatment to date limited to just two patients in the United States. Individuals typically require ongoing care from a multidisciplinary team, including paediatricians, neurologists, ophthalmologists, physiotherapists, occupational therapists, and speech therapists (Nair et al. 2023). Assistive technologies, mobility aids, and educational supports are often required, and care needs tend to increase over time as neurodegeneration progresses with age. As a result, individuals with KAND rely heavily on family members for assistance with daily activities, placing substantial physical, emotional and financial demands on caregivers (Nair et al. 2023).

KAND has been recently classified within childhood dementia, a group of genetically heterogeneous neurodegenerative conditions characterised by progressive cognitive and developmental decline and reduced life expectancy (Nunn et al. 2002). Families affected by childhood dementia face many shared challenges (Nevin et al. 2023). Symptoms often emerge unpredictably and progress at variable rates, creating ongoing uncertainty and complicating long-term planning (Mori et al. 2019; Palacios-Ceña et al. 2019; Porter et al. 2021). Parents frequently encounter bureaucratic barriers when attempting to access supports, as publicly funded systems are often ill-equipped to respond to their child’s complex and changing needs (Krantz et al. 2022; Somanadhan and Larkin 2016). Behavioural symptoms, including aggression and hyperactivity, may worsen with age, are often stigmatised, and can increase caregiver anxiety and social isolation (Hoffmann et al. 2020; Nevin et al. 2023; Porter et al. 2021). Uncertainty surrounding long-term care, education, and assistive technologies, combined with escalating caregiving and financial demands, further compound this burden. In Australia, families may access government-funded disability supports through the National Disability Insurance Scheme (NDIS); however, even with this funding, children with complex developmental conditions remain unable to participate in daily life without profound levels of unpaid care and parental support (O’Neill et al. 2024). This highlights important parallels for understanding support requirements in KAND. The challenges faced by Australian families reflect broader international patterns, with families in the USA and Europe similarly reporting substantial care burdens and financial strain associated with childhood neurogenerative conditions (Delval et al. 2026).

The extreme rarity and distinct clinical features associated with KAND introduce additional complexity. Progressive visual impairment, severe epilepsy, and neuropathic pain may further complicate care and long-term planning, particularly in the context of limited access to condition-specific expertise and information. Consequently, findings from the childhood dementia literature are informative but insufficient to fully characterise the specific clinical and psychosocial challenges faced by the KAND community. This gap limits clinicians, support services, and policymakers in understanding the psychosocial, caregiving, and financial challenges faced by these families, many of which are not apparent from the clinical data alone. This study addresses this gap through in-depth interviews with individuals and families affected by KAND. It explores their healthcare experiences, impacts on family life, unmet support needs, and coping strategies used, with the aim of informing more responsive clinical care, support services, and policy development.

## Methods

### Study design

The study was co-designed with the KIF1A Australia Foundation (KIFA.AU), a parent-led non-profit organisation (KIF1A Australia 2025), in response to affected families identifying a need for research into their experiences of KAND. This research emerged from an online information session with members of the KAND community, facilitated by KIF1A.AU, during which participants provided feedback on research priorities. This feedback informed the development of the research question and interview guide (see Supplementary Material).

### Participants and recruitment

Participants were recruited through convenience sampling in partnership with KIF1A.AU. Eligible participants were adults aged 18 years and over, residing in Australia or New Zealand, with the ability to communicate in English. The study included individuals with a genetic diagnosis of KAND, their primary caregivers, and immediate family members. Exclusion criteria were an inability to provide informed consent and requiring an interpreter, since translation resources were unavailable for the project. All participants were emailed a copy of the Patient Information and Consent Form (Supplementary Material) prior to participation. The researcher answered all questions raised by participants before written consent was obtained via email by the lead researcher. All procedures followed were in accordance with ethical standards and approved by the Human Research Ethics Committee of The Royal Children’s Hospital (HREC 113655).

### Data collection

This exploratory qualitative study used semi-structured interviews to explore the experiences of individuals and families affected by KAND. The interviews followed a semi-structured interview guide that focused on four key areas: diagnosis experience, daily challenges, unmet support needs, and coping strategies. The interview guide was designed by the research team, consisting of qualitative, KAND, and health research experts with extensive experience in appropriate interview design. All interviews were conducted over Zoom, audio recorded, transcribed verbatim, and further edited for accuracy. Participants were welcomed to pause, stop or withdraw from the interviews at any time. Prior to interviews, participants completed a voluntary demographic survey (Supplementary Material). All participants received a voucher of $50 as a thank you for their time.

### Data analysis

The collected interview data were analysed using inductive content analysis (Elo and Kyngäs 2008) with NVivo qualitative software used to organise and code interview transcripts. This analytic approach was selected given the limited existing research, the exploratory nature of the study, and its practical focus.

Following coding of the first four transcripts, an initial coding framework was refined collaboratively by our research team, which included experts on KAND and qualitative research. Two transcripts were independently coded by the authors KN and TD, and subsequent coding and analysis meetings were held to ensure that codes and subcategories were well supported by the data. The process of reflexivity was actioned throughout the analytic process whereby the team regularly discussed and questioned potential bias and assumptions from our own experiences that could risk influencing the data findings.

## Results

Seventeen participants took part in this study, with a total of 15 interviews conducted. In two cases, interviews were conducted jointly (e.g. a mother and father), accounting for the difference between the number of participants and interviews. Interviews ranged from 37 to 76 min in length (mean: 53 min). Participant demographic information based on survey data is presented in Table 1. More detailed demographic information can be found in the Supplementary Material. All participants were parents of individuals diagnosed with KAND; however, one participant was also living with the condition herself. This participant was supported by a family member and had adequate language proficiency and cognitive capacity to enable participation and provision of informed consent.

**Table 1:** Participant demographic information

Characteristics	Respondents (*n*=17)
Age of participant	
30-39	2
40-49	12
50-59	0
60-69	1
70-79	1
Age of child with KAND	
0-9	7
10-19	7
20-29	2
30-39	1
Gender of participant	
Male	3
Female	14
Relationship status	
Single	1
In a de facto relationship	1
Married	12
Separated	1
Divorced	2
Annual household income	
Below national median	4
Above national median	10
Prefer not to say	3
Geographic location	
City/metropolitan	9
Regional/rural	8

Note: Participants were categorised based on the Australian median household income as reported by the Australian Bureau of Statistics (2022)

**Fig. 1:**
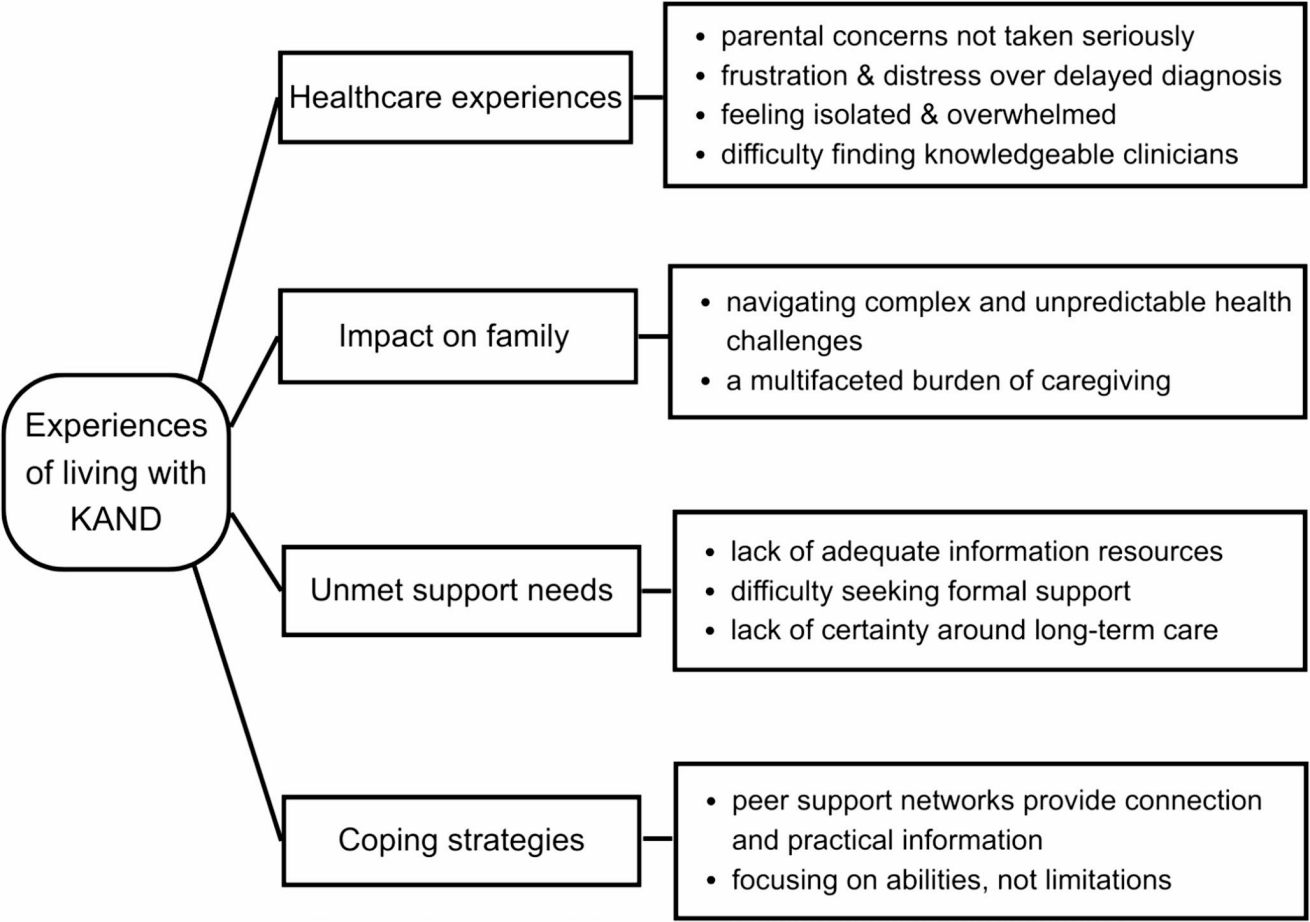
Analysis framework illustrating the organisation of emerged categories and subcategories identified through inductive content analysis, describing the experiences of individuals and families affected by KAND

Analysis of the interview data was organised around four broad categories corresponding to key topics explored in the interviews: (1) healthcare experiences; (2) impact on family; (3) unmet support needs; and (4) coping strategies (Fig. 1). The sub-categories, and illustrative quotes presented in the following section. Additional quotes can be found in Supplementary Material (Table of Additional Quotes).

### Healthcare experiences

Families described healthcare experiences characterised by delayed responses, limited clinician knowledge, and inadequate support, resulting in sustained frustration and distress. Early parental concerns were frequently minimised or dismissed, with symptoms attributed to personality traits or normal developmental variation rather than prompting further investigation.

*“A lot of the specialists we had seen were just basically saying ‘It’s fine. Don’t worry about it’. In fact*,* the paediatrician we saw said it was his personality.” Participant 3*.

Requests for additional assessments, including genetic testing, were commonly met with resistance, resulting in prolonged diagnostic delays spanning months or years. These delays were particularly distressing in hindsight, as parents reflected on missed opportunities for early intervention and more appropriate symptom monitoring.

*“We were told that it was cerebral palsy*,* so we started treating it like it was cerebral palsy…If we had have known that it was degenerative back then*,* we probably would have done more early intervention.” Participant 6*.

Receiving an ultra-rare diagnosis was described as overwhelming and isolating. Limited clinician familiarity with KAND, poor coordination across services, and minimal referral to peer support compounded these challenges. Diagnostic consultations were often perceived as poorly managed, with large volumes of complex information delivered during emotionally distressing encounters. Parents emphasized the importance of empathy, clear communication, and emotional attunement.

*“It’s more important to just connect with the family…instead of dumping information about KIF1A” Participant 2*.

Ongoing care was further complicated by difficulties accessing healthcare professionals with knowledge of KAND. Parents described repeatedly encountering clinicians unfamiliar with the condition, reinforcing uncertainty and isolation.

*“I told the doctor she’s got KIF1A and he said I don’t know what that is. And every doctor I mentioned*,* no one knows anything.” Participant 9*.

Families also reported challenges identifying appropriate services and support pathways, highlighting the need for clearer guidance and consolidated resources (e.g. a database of clinicians knowledgeable about KAND). Similarly, clinicians would have benefited from condition-specific resources to support families, given that they had likely not encountered the condition.

### Impacts on the family

Families described the impact of KAND on family life as profound and multifaceted, affecting both the individual’s quality of life and overall family functioning. Behavioural and sensory sensitivities were consistently identified as among the most challenging features. Hypersensitivity to noise and light were frequently reported to trigger behavioural outbursts, limiting families’ ability to participate in everyday activities and increasing stress.

*“You can’t take her to the shopping centre because she just screams…You go to a shopping centre food court and someone moves the chair*,* she screams.” Participant 9*.

Communication difficulties were also prominent. While many individuals with KAND demonstrated strong receptive language, expressive impairments led others to underestimate their cognitive abilities, contributing to frustration and social exclusion:

*“People just think that she doesn’t understand…and they just move on and will talk over the top of her…I think that’s very*,* very frustrating for her.” Participant 1*.

Combined with mobility challenges, visual impairment, and poor balance, these challenges created ongoing safety risks and necessitated constant supervision. One parent described recurrent falls as life-threatening, stating, *“She’s lucky to be alive.” Participant* 12.

Caring for an individual with KAND imposed substantial financial, administrative, psychological and relational burdens. Despite access to government funding, indirect costs – particularly lost income and time spent navigating support systems – were significant. Parents described extensive time commitments for administrative tasks, particularly relating to the National Disability Insurance Scheme (NDIS):

*“We spend probably 10 hours a week on [the NDIS]*,* and have done for the last two years*,* trying to get the right supports for our son.” Participant 13*.

Parents also reported considerable psychological distress, including loneliness, emotional isolation, and a sense of disconnection from the ‘typical’ world. These feelings were intensified by the rarity of KAND, limited social understanding, and the unrelenting demands of caregiving:

*“I was really lost because I felt like a lone ranger…I felt like it’s me and [my daughter] against the world*,* but [my daughter] is ignoring me.” Participant 2*.

### Unmet support needs

Parents identified significant systemic barriers to obtaining adequate support, including limited information at diagnosis and poorly defined pathways to formal assistance. Information provided by clinicians was often described as either insufficient or overly technical, offering little practical guidance for daily care. Participants expressed a strong preference for accessible, practical resources that reflected the heterogeneity of KAND, including clear prognostic information and guidance on available supports.

*“I want to know the practical side…I want to know what we can actually do and what other parents have done and tried*,* and what has worked.” Participant 4*.

Accessing formal supports, particularly through the NDIS, was described as especially challenging. The ultra-rare nature of KAND meant the condition was often poorly recognised, requiring sustained parental advocacy to secure appropriate supports. Parents reported that limited understanding of KAND’s clinical variability within the National Disability Insurance Agency (which oversees the NDIS program) resulted in standardised support plans that failed to reflect the severity and complexity of their child’s individual needs:

*“The National Disability Insurance Agency… don’t fully understand or want to know or understand what these kids are like or how different one child can be from the next…They are putting everybody in the same group.” Participant 6*.

Concerns about long-term care were also substantial. Parents described uncertainty regarding future care arrangements, particularly in scenarios where they might no longer be able to provide primary care. A lack of clear pathways or guidance contributed to ongoing anxiety around their child’s future.

*“If anything ever happened to [my husband] and I*,* what kind of home would [my son] go into? We don’t know. The National Disability Insurance Agency can’t tell us.” Participant 6*.

### Coping strategies

Families described multiple strategies that helped them to cope with challenges in caring for a child with KAND. Peer support networks, including KIF1A.AU (Australia) and KIF1A.ORG (USA), were highly valued and often perceived as more informative and practical than clinician-provided information. Parents highlighted the importance of shared lived experience; particularly opportunities to connect with families of children with similar genetic variants.

*“I learnt the most information just connecting with other families and looking at their children*,* maybe with the same variant as [my daughter]*,* and then seeing how they’re progressing.” Participant 9*.

Maintaining hope by focusing on their child’s strengths rather than limitations was another key coping strategy. Parents described celebrating developmental gains, even when progress was slow, and emphasising positive traits such as determination, sociability, and resilience.

*“We just keep focusing on the things that he can do rather than the things that he can’t.” Participant 15*.

Many parents expressed gratitude for improvements attributed to early and consistent therapeutic interventions, which they felt enhanced their child’s communication and quality of life.

*“[My son] was practically non-verbal at one point. Very early on in the piece*,* I worked very closely with a speech therapist…and that enabled us to get [my son] to a point where he could tell us how he felt.” Participant 6*.

## Discussion

This study provides the first exploration into the experiences of individuals and families affected by KAND. We recruited a substantial proportion of families affected across Australia and New Zealand (representing 16 of the 28 individuals genetically diagnosed with KAND), capturing a wide range of experiences. Participants described prolonged and distressing diagnostic journeys, fragmented healthcare experiences, substantial caregiving burdens, and persistent unmet support needs, collectively highlighting the profound impact of KAND on family life.

Many of the challenges reported mirror those documented in childhood dementia and other neurodevelopmental conditions, including poor care coordination (Bose et al. 2019), uncertainty regarding long-term care (Nevin et al. 2023), high caregiving demands (Williamson 2019), behavioural management difficulties (Nevin et al. 2023), and barriers accessing disability funding (O’Neill et al. 2024). These parallels suggest that families affected by KAND encounter systemic barriers similar to those observed across childhood dementia, reflecting broader deficiencies in integrated rare disease care. However, these families may face additional challenges due to the limited availability of information resources and restricted access to clinicians and support services with relevant expertise. Consistent with recommendations from the Childhood Dementia Initiative (2020), our findings support cross-condition approaches to research, service delivery, and support programs, which may improve efficiency and equity while enabling opportunities for collective progress (Elvidge et al. 2025). At the same time, interventions tailored specifically to KAND remain necessary alongside broader efforts to improve care for childhood dementia.

Several challenges appear distinctive to KAND or are intensified by its ultra-rare nature. In particular, sensory hypersensitivities and environmentally triggered behavioural outbursts were prominent yet remain poorly described in the existing literature. Parents frequently linked behavioural distress to overstimulation from light, sound, or crowded environments, drawing parallels with autism spectrum disorder, a recognised but underexplored feature of KAND. These symptoms substantially limited family participation in social and community activities and contributed to parental stress. Recognising these challenges explicitly in clinical consultations may better equip families to anticipate and manage them effectively.

Physical impairments, including progressive mobility limitations and vision loss, further intensified caregiving demands, often necessitating continuous supervision. Together, these cognitive, behavioural and physical challenges underscore the need for clearer guidance for clinicians and families on common symptom trajectories and their real-world implications. Consistent with previous literature (Saini et al. 2024), therapeutic interventions were described as critical for maintaining function and independence. Communication difficulties emerged as a particularly significant concern, contributing to frustration from both individuals with KAND and their families, consistent with observations by Morison et al. (2025). Parents reported meaningful improvements with speech therapy, including progression from minimal verbal communication to functional speech in some cases, reinforcing the importance of timely and sustained access to therapies despite the degenerative nature of the condition.

The overall caregiving burden described by parents was extensive and all-encompassing. This burden was further compounded by limited clinician familiarity with KAND, requiring parents to assume roles not only as caregivers but also as advocates, care coordinators, and KAND experts in their own right, including educating clinicians involved in their child’s care. The cumulative administrative, emotional, and cognitive labour, combined with restricted access to respite services, contributed to exhaustion, guilt, and reduced opportunities for self-care. These findings reinforce the need for greater access to professional support and respite to protect caregiver wellbeing and improve overall family functioning.

Families also encountered substantial barriers to diagnosis and ongoing care, consistent with patterns observed across rare diseases (Lopes et al. 2018). Diagnostic delays were frequently attributed to clinician unfamiliarity, misdiagnosis (most commonly as cerebral palsy), and delayed recognition of the significance of early developmental concerns. Parents often described their concerns being initially minimised or attributed to normal developmental variation or personality traits before further investigation was pursued. Given the benefits of early diagnosis for care planning, increased awareness of KAND is essential, particularly in cases initially suspected to be cerebral palsy. Although inclusion of *KIF1A* on diagnostic gene panels, including the Australian PanelApp, represents progress, this must be complemented by accessible clinician resources to translate genomic advances into timely diagnoses.

The delivery of the diagnosis itself was often described as distressing, characterised by overwhelming or insufficient information and a lack of referral to peer support. While some educational materials on KAND exist, including a factsheet on the symptoms of KAND and basic supports (Centre for Genetics Education 2021) and on speech and language (Translational Centre for Speech Disorders 2025), participants identified persistent gaps in practical guidance, particularly regarding navigating support services, accessing entitlements, and understanding the roles of different healthcare professionals. These findings emphasise the value of co-designing resources with families to ensure relevance to daily life.

Access to clinicians with knowledge or experience in KAND was another prominent unmet need. While it is unrealistic for all clinicians to be familiar with KAND, families stressed that clinicians should, at a minimum, provide clear, empathetic and practical guidance to newly diagnosed families, including referral to peer support networks such as KIF1A.AU. To support clinicians encountering unfamiliar rare conditions, readily accessible, disease-specific resources should be available when needed. Genomic education for clinicians is also important as genomic testing becomes more widely integrated into clinical practice, facilitating the diagnosis of rare diseases that may previously have remained undiagnosed. Beyond educational resources, participants strongly emphasised the potential value of a centralised network or registry of clinicians with relevant expertise or interest. Such a model could improve referral pathways, reduce the burden on families, and enhance continuity of care. Furthermore, while some delays in diagnosis may be unavoidable due to the rarity of KAND, broader support organisations such as SWAN (Syndromes Without A Name) may provide valuable interim support for families navigating uncertainty prior to receiving a diagnosis.

Navigating the NDIS was a major challenge for many families. Although difficulties with the NDIS are well documented in other conditions, such as cerebral palsy (O’Neill et al. 2024), they appeared particularly pronounced in KAND due to phenotypic heterogeneity and misunderstandings regarding the value of therapies in degenerative conditions. Despite formal recognition of KAND by the NDIS, greater workforce education is required to ensure funding decisions reflect the functional benefits of early intervention and ongoing therapy.

The demographic profile of participants suggests inequities in access to diagnosis, with an overrepresentation of highly educated and financially secure families, some with healthcare backgrounds. This aligns with broader rare disease literature and suggests that individuals lacking such resources may remain undiagnosed or misdiagnosed. There may be a significant number of individuals in Australia, New Zealand, and indeed globally, who have been misdiagnosed or are yet to receive a KAND diagnosis.

Families employed a range of coping strategies, most notably engagement with peer support networks and positive reframing. The peer support provided not only psychological benefits by fostering connection and reducing isolation, but also practical, experience-based advice. While dedicated peer support organisations for KAND already exist, many families were unaware of these organisations until much later in their journey. This disconnect indicates a systemic failure: the provision of information regarding peer support organisations is fragmented and primarily disseminated by the foundations themselves rather than integrated into clinical pathways. Embedding these referrals into standard practice at the point of diagnosis could have a meaningful impact on families affected by KAND, and by extension, rare diseases more broadly.

While participants strongly valued diagnosis-specific peer support through organisations such as the KIF1A Australia Foundation and KIF1A.ORG, our findings also raise broader questions about how support communities are conceptualised in rare neurodevelopmental and neurodegenerative conditions. Many challenges described by families, including fragmented healthcare, social isolation, caregiving burden, and uncertainty around long-term prognosis, are shared across childhood dementias and other severe neurodevelopmental disorders. As such, broader rare disease communities may offer important psychosocial support and opportunities for shared advocacy across conditions.

However, our findings also indicate that diagnosis-specific communities remain particularly valuable in ultra-rare disorders such as KAND, where families often seek highly specific experiential knowledge regarding symptom progression, potential therapies, prognosis, and emerging research. In the era of precision medicine, molecular diagnoses are also increasingly shaping access to patient registries, clinical trials, and future targeted therapies. This highlights a networked model of support in which families can engage with both diagnosis-specific and cross-condition communities as complementary and interconnected resources, collectively strengthening psychosocial support, advocacy, and research engagement in rare disease.

### Future directions and implications

Most of the data from this study was obtained from carers rather than individuals directly affected by KAND. While this reflects the reality that many individuals with KAND experience significant intellectual disability, it frames the findings through a predominantly caregiver lens. Future research should seek to incorporate adapted communication methods to ensure that individuals with varying levels of disability are able to contribute to research (Strnadová et al. 2023).

Targeted education and the development of accessible, patient-centred information resources is of great importance. Raising awareness about KAND should focus on healthcare professionals that are most likely to encounter affected children, including general practitioners, paediatricians, neurologists, geneticists, and genetic counsellors. Research exploring the experiences of healthcare professionals providing care for individuals with KAND may inform strategies to better support clinicians and improve healthcare delivery. At a more systemic level, there is a strong need for education of the NDIS workforce in terms of variability in clinical presentations, and the continuing importance of therapies and early intervention despite a degenerative disease trajectory. Grouping KAND under the umbrella term of ‘childhood dementia’ may facilitate understanding among clinicians and policymakers, allowing funding and services to be allocated more fairly (Elvidge et al. 2025).

Future research should investigate the economic burden of KAND, as well as strategies to improve clinician knowledge and communication. Expanding the study to include international families would provide valuable insights into how these findings apply across different healthcare systems and cultural contexts.

These findings underscore the urgent need to better support families affected by KAND. Key priorities include establishing a centralised network or registry of clinicians with relevant expertise, providing more practical and comprehensive information resources, improved access to NDIS funding – including increased respite hours – and ensuring diagnoses are delivered with compassion, alongside clear referral pathways to peer support organisations. Greater recognition of KAND under the umbrella of childhood dementia may also enhance clinical understanding and awareness.

More broadly, this study contributes to our understanding of childhood dementia and rare disease, while emphasising the unique challenges faced by families living with KAND. Addressing these unmet needs has the potential to improve diagnostic pathways, strengthen support services, and enhance quality of life for affected individuals and their families. Importantly, these insights also help guide research priorities and clinical practice as the field moves toward to the development of effective treatments, and ultimately a cure for KAND.

## Supplementary Information

Below is the link to the electronic supplementary material.

Supplementary Material 1

Supplementary Material 2

## Data Availability

The datasets generated and/or analysed during the current study are available from the corresponding author on reasonable request.
