# Supplementary material for: “I felt like a lone ranger”: experiences of Australian families living with KIF1A-Associated Neurological Disorder
Source: J Community Genet. 2026 Jun 8;17(3):73. doi: 10.1007/s12687-026-00908-5 (PMC13243148; doi:10.1007/s12687-026-00908-5)
Supplement: Supplementary file 1 — Supplementary Material 1 [file 12687_2026_908_MOESM1_ESM.docx]

**SUPPLEMENTARY INFORMATION – JOURNAL OF COMMUNITY GENETICS**

**“I Felt Like a Lone Ranger”: Experiences of Australian Families Living with *KIF1A*-Associated Neurological Disorder**

Kara Miwa-Dale, Kimberley Norman, Belinda Dawson-McClaren, Jeanette Harris, Wendy A. Gold, Trang T. Do, Simranpreet Kaur

Corresponding author: Dr. Simranpreet Kaur^1,2^: [simran.kaur@unimelb.edu.au](mailto:simran.kaur@unimelb.edu.au)

^1^ Department of Paediatrics, The University of Melbourne, Melbourne, VIC, Australia. ^2^ Brain and Mitochondrial Research Group, Murdoch Children's Research Institute, Royal Children's Hospital, Melbourne, VIC, Australia

**a) Interview Guide**

**Introduction**

Introductions (participant and researcher)

Explain the project by summarising the key points in the Participant Information Sheet and reiterate to the participant their rights again (ie they can stop at any time, no questions asked)

Explain what will happen in their time with you.

Answer any questions the participant has before recording.

Make sure consent is signed and returned to the research team.

Turn on Audio/ Video Recording – ask consent to start the recording and record the verbal consent if the participant has not returned a signed consent form.

A range of issues to be covered in the interview:

- Context and experience of the diagnostic process
- Interaction with the health system
- Interaction with peers/peer group
- Impacts of KAND on their everyday life
- Expectations for service improvement and future support
- Suggestion for future research

**The interview**

When the participant is ready, begin with the first question:

***Individual with KAND***

Question 1: “Can you describe your experience with the diagnosis of KAND?”

Prompts:

- When and how did you first learn about the diagnosis?
- What did you know about the condition when you received the diagnosis?
- How was your experience with healthcare providers throughout the diagnosis process?
- Is there anything about the condition you wish you had understood better?
- What were the challenges you faced during the diagnostic journey? Was there anything that made the journey easier?
- Are there any services or resources that you found particularly helpful throughout the diagnostic journey?

Question 2: “Can you tell me about your experiences living with KAND?”

Prompts:

- “How has KAND impacted your day-to-day life?”
- “What are the challenges/difficulties you face when living with KAND?”
- “How did you find out about the support available to you (from family members, peer-support network, social network or from healthcare providers?”
- “Have you encountered any difficulties in accessing support?”

Question 3: “What kind of support services do you think would benefit individuals affected by KAND?

- How do you think existing support services could be improved for people living with KAND?
- What are the most important aspects of a support service for individuals affected by KAND?
- How could support services be made more accessible or tailored to the needs of individuals with KAND?

Question 4: “What suggestions do you have for future research to better understand KAND and improve health outcomes?”

- What areas of KAND do you think need more attention in research?
- What types of studies or research approaches do you think would be most beneficial for better understanding KAND?
- How do you think research could help improve the quality of life for those affected by KAND?

***Family of person with KAND***

Introductory question: Tell me a bit about your child with KAND – what are they like?

Question 1: “Can you describe your experience when your child was diagnosed with KAND?”

- “When and how did you first learn about the diagnosis?”
- “What did you know about the condition when you received the diagnosis?”
- How was your experience with healthcare providers throughout this process?
- What do you wish you could have known more about regarding the condition?
- “What were the challenges you faced during the diagnostic journey? Was there anything that made the process easier?
- “Are there any services or resources that you found particularly helpful throughout the diagnostic journey?”

Question 2: “How has having a family member with KAND affected your daily life?”

Prompts:

- “What has changed in your family routine since the KAND diagnosis?”
- “What are the challenges in providing care to your family member with KAND?” OR What are the challenges you experience when caring for your family member with KAND?
- “How do you cope with those challenges?”
- “Have you received any support (from peer-support network, social network or from healthcare providers?”

Question 3: “What kind of support services do you think would benefit families affected by KAND?

Question 4: “What suggestions do you have for future research to better understand KAND and improve health outcomes?”

**Closing**

Is there any topic or issue you would like to discuss to help me understand more about your experience living with KAND/providing care to a person with KAND?

Thank the participant for their time and inform them of any follow-up activities with the research study.

Stop recording

**b) Patient Information and Consent Form**


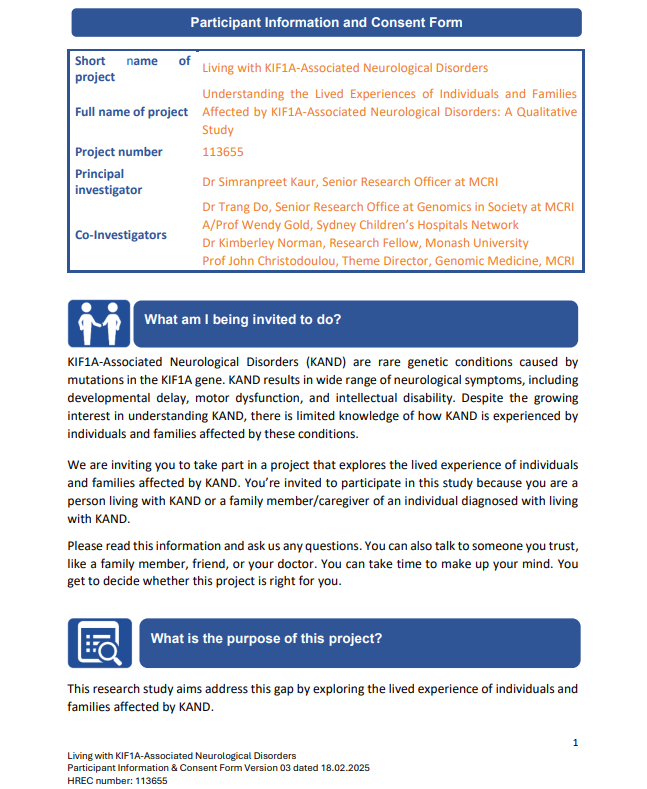


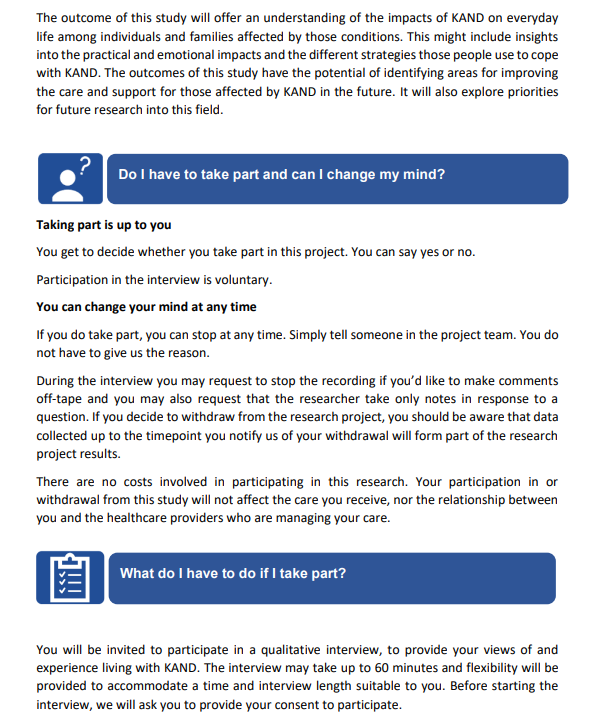


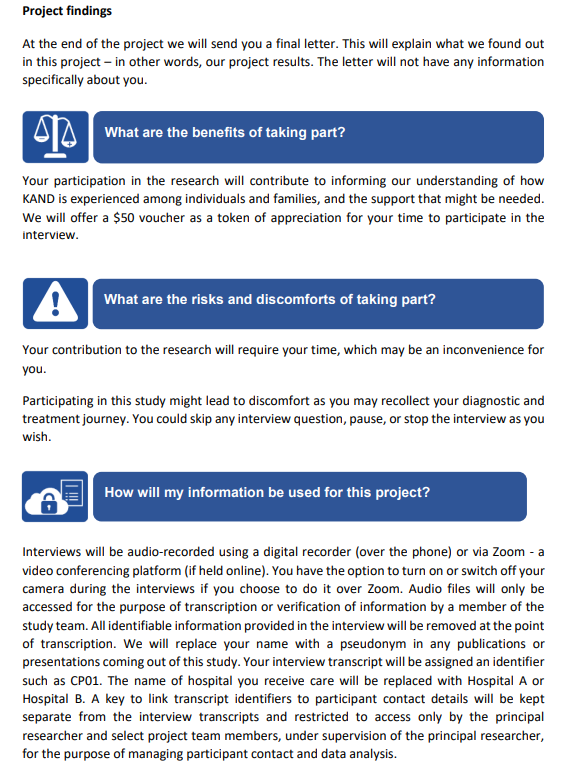


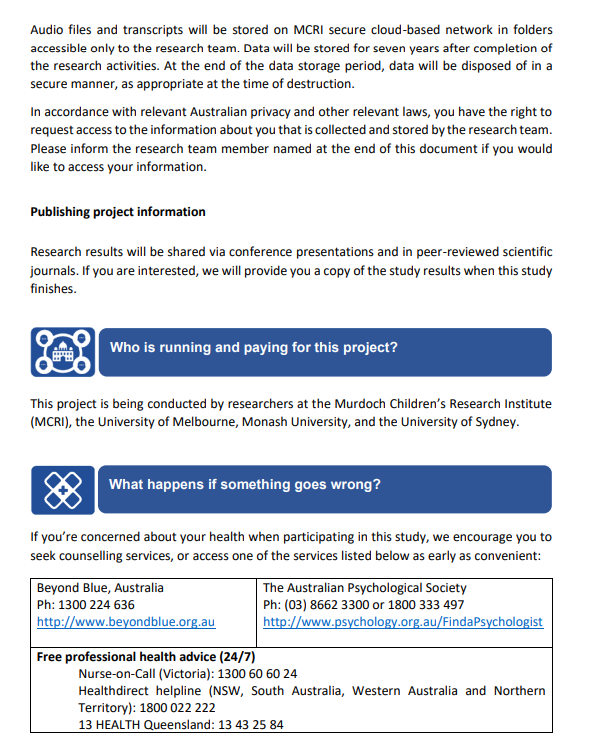


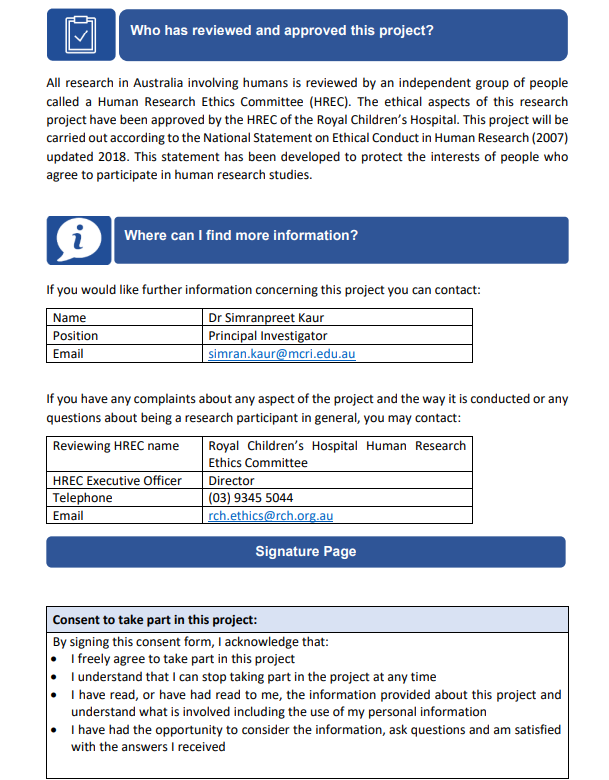


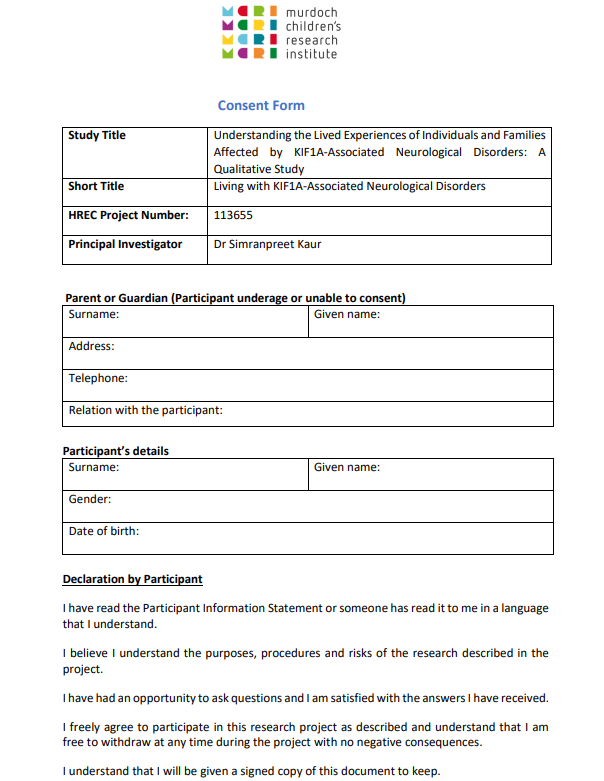


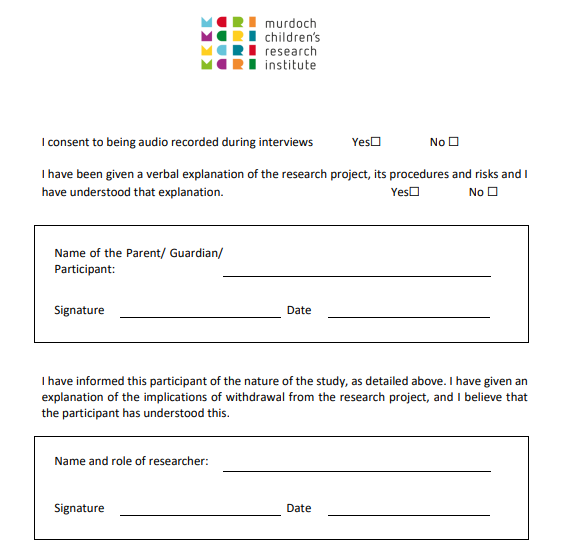


**c) Demographics survey**


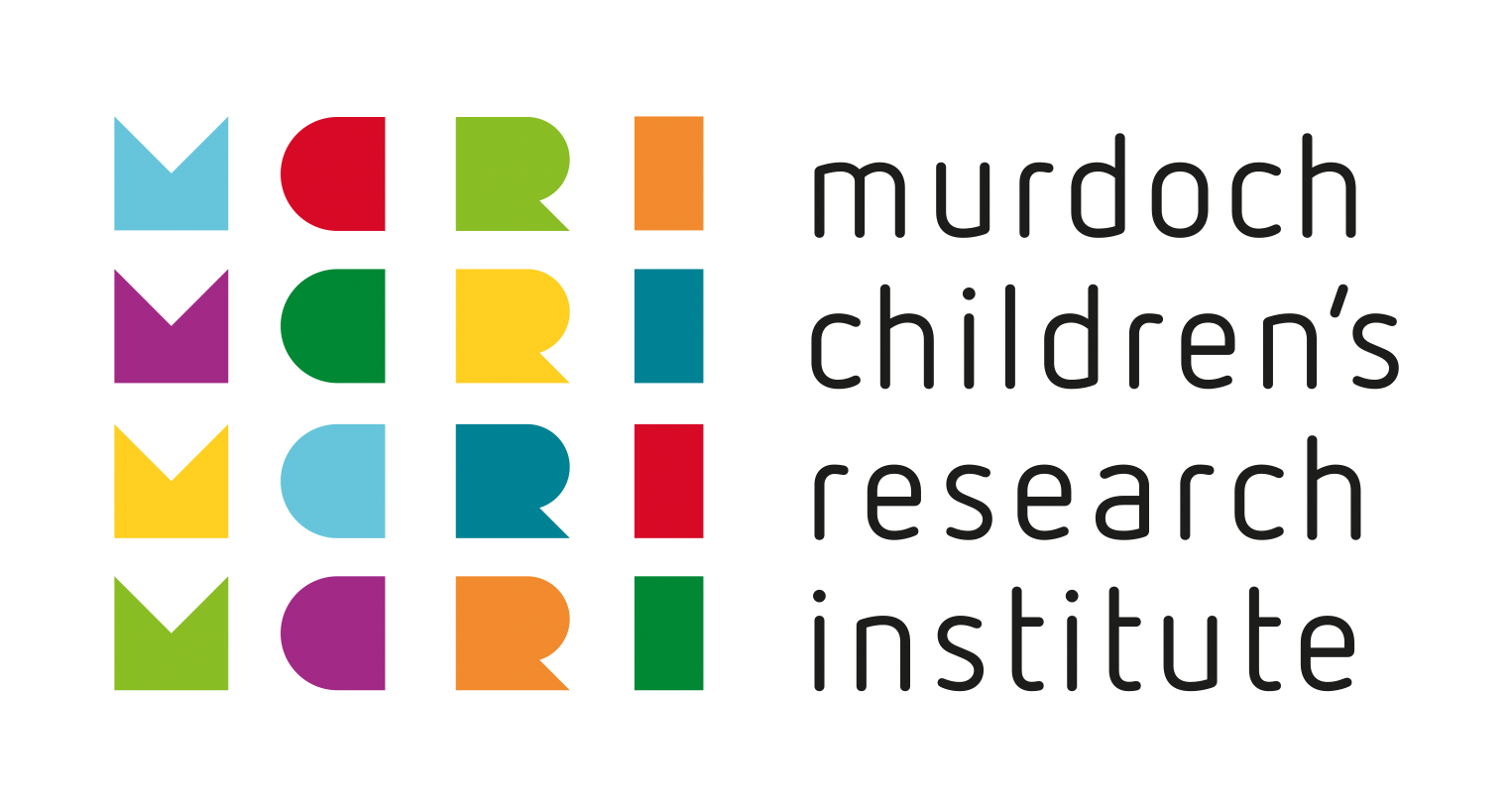


**Demographic Survey**

| **Study Title** | Understanding the Lived Experiences of Individuals and Families Affected by KIF1A-Associated Neurological Disorders: A Qualitative Study |
| --- | --- |
| **Short Title** | Living with KIF1A-Associated Neurological Disorders |
| **HREC Project Number:** | 113655 |
| **Principal Investigator** | Dr Simranpreet Kaur |

**Name of the participant: ____________________**

Q1. What is your age?

Q2. What gender do you identify as?

Q3. What is your highest level of education?

No formal education

Primary school

Some secondary education (up to Year 9)

Completed Year 10 (Junior secondary)

Completed Year 12 (Senior secondary)

TAFE/trade certificate or diploma

University undergraduate degree (e.g. Bachelor’s)

Postgraduate Degree (e.g. Masters, PhD)

Other:

Prefer not to say

Q4. What is your occupation?

Q5. Where do you live?

1. Which state or territory do you live in?

New South Wales

Victoria

Queensland

South Australia

Western Australia

Tasmania

Australian Capital Territory

Northern Territory

Prefer not to say

1. What kind of area do you live in?

City/metropolitan area

Regional/rural area

Remote area

Prefer not to say

Q5. What is your marital/relationship status?

Single

In a relationship (not living together)

In a de facto relationship (living together, not married)

Married

Separated (but not divorced)

Divorced

Widowed

Other: Click or tap here to enter text.

Prefer not to say

Q6. How many people are there in your household? Click or tap here to enter text.

Q7. Which income range best represents your total household income for the past year?

Less than $75,000

$75,000 - $100,000

$100,000 - $150,000

$150,000 - $200,000

Over $200,000

Prefer not to say
